# Left Ventricular Hypertrophy: Roles of Mitochondria CYP1B1 and Melatonergic Pathways in Co-Ordinating Wider Pathophysiology

**DOI:** 10.3390/ijms20164068

**Published:** 2019-08-20

**Authors:** George Anderson, Gianluigi Mazzoccoli

**Affiliations:** 1 CRC Scotland & London, London E14 6JE, UK; 2 Division of Internal Medicine and Chronobiology Unit, Department of Medical Sciences, Fondazione IRCCS “Casa Sollievo della Sofferenza”, San Giovanni Rotondo, 71013 Foggia, Italy

**Keywords:** left ventricular hypertrophy, mitochondria, CYP1B1, melatonin, N-aceytlserotonin, MAPK, sirtuin, gut microbiome, treatment, aryl hydrocarbon receptor

## Abstract

Left ventricular hypertrophy (LVH) can be adaptive, as arising from exercise, or pathological, most commonly when driven by hypertension. The pathophysiology of LVH is consistently associated with an increase in cytochrome P450 (CYP)1B1 and mitogen-activated protein kinases (MAPKs) and a decrease in sirtuins and mitochondria functioning. Treatment is usually targeted to hypertension management, although it is widely accepted that treatment outcomes could be improved with cardiomyocyte hypertrophy targeted interventions. The current article reviews the wide, but disparate, bodies of data pertaining to LVH pathoetiology and pathophysiology, proposing a significant role for variations in the N-acetylserotonin (NAS)/melatonin ratio within mitochondria in driving the biological underpinnings of LVH. Heightened levels of mitochondria CYP1B1 drive the ‘backward’ conversion of melatonin to NAS, resulting in a loss of the co-operative interactions of melatonin and sirtuin-3 within mitochondria. NAS activates the brain-derived neurotrophic factor receptor, TrkB, leading to raised trophic signalling via cyclic adenosine 3′,5′-monophosphate (cAMP)-response element binding protein (CREB) and the MAPKs, which are significantly increased in LVH. The gut microbiome may be intimately linked to how stress and depression associate with LVH and hypertension, with gut microbiome derived butyrate, and other histone deacetylase inhibitors, significant modulators of the melatonergic pathways and LVH more generally. This provides a model of LVH that has significant treatment and research implications.

## 1. Introduction

Cardiac hypertrophy refers to a thickening of the walls of a ventricle, usually the left (LVH), but sometimes the right (RVH), and occasionally both. This can be adaptive, as in aerobically trained athletes or in pregnancy in response to increased blood flow/volume. However, it can also occur in response to heightened blood pressure or as the consequence of a heart attack-induced ventricular remodelling or from valvular or myocardium defects. Generally, adaptative and pathologic remodelling have differentiated consequences, based on the overlapping, but distinct, cellular processes that are engaged. Factors associated with metabolic dysregulation, including stress and diabetes, have an increased risk of hypertension and LVH.

Given that the cardiac muscle cell has limited proliferative ability, modification of the heart muscle mass and volume is driven by hypertrophic remodelling, whereby cardiomyocytes increase their volume, and alter their shape and genes expressed [1]. This is done in association with alterations in the cytoskeleton, mitochondria, small GTPases and in the extracellular matrix (ECM) [2]. Suboptimal mitochondria functioning is a core aspect of LVH, with an increase in the levels of glucose utilization as an energy substrate, paralleling the mitochondria changes evident in many cancers. A number of consequences arise from alterations in mitochondria functioning, including increased reactive oxygen species (ROS) and alterations in energy production [3]. Peroxisome proliferator-activated receptor gamma coactivator 1-alpha (PGC–1α) is the master regulator of mitochondria metabolism, with PGC-1α being decreased in LVH cardiomyocytes [4]. Such data hints at core changes in mitochondria functioning that act to drive and co-ordinate the wide array of biochemical changes evident in LVH [5,6]. Given that mitochondria form about 30% of the volume of cardiomyocytes [7], it is clear that alterations in mitochondria functioning, shape and size are crucial to wider cellular organization.

Many of the changes associated with LVH are also evident in proliferative conditions, such as endometriosis and a wide array of cancers [8,9]. Recent works suggest that alterations in mitochondria functioning, particularly the regulation of the melatonergic pathways, may be a core aspect of how changes in mitochondria drive the co-ordinated pathophysiological processes underpinning these diverse medical disorders [8,9]. The current article reviews how aryl hydrocarbon receptor (AhR)-induced cytochrome P450 (CYP)1B1 suppresses mitochondria melatonin production, thereby initiating the co-ordinated cellular changes evident in LVH. Such a frame of reference acts to integrate the large collection of previously disparate data on LVH pathophysiology. The key aspects of the biological underpinnings of LVH are reviewed first.

## 2. LVH Pathophysiology

The pathoetiology of LVH is intimately associated with increased blood pressure and therefore with the array of different factors associated with hypertension, including the various manifestations and consequences of stress, obesity and diabetes. Although treatment is primarily geared to blood pressure control, which can reverse many of the hypertrophic changes in LVH, it is widely accepted that treatment more directly targeted to LVH pathophysiology would improve patient outcomes. LVH is associated with a number of changes, including the mitogen-activated protein kinase (MAPK) pathways, CYP1B1, the AhR, oxidative stress, immune-inflammation, the kynurenine pathways, gut dysbiosis, sirtuins, microRNAs, 14-3-3 proteins and mitochondria functioning. These are looked at in more detail, before being integrated into a model that highlights the role of the melatonergic pathway in mitochondria, which is proposed to integrate the core pathophysiological features of LVH.

### 2.1. Mitogen-Activated Protein Kinase (MAPK) Pathways

Numerous intracellular pathways have been associated with LVH, as with many proliferative conditions [8], including activation of the MAPK pathways, which are strongly associated with both exercise-induced and pathophysiological cardiac hypertrophy [1,10,11,12,13]. The MAPK pathways are important regulators of many physiological and pathophysiological processes, with roles in cell proliferation and stress responses. The protein 38 (p38) MAPK branch of the MAPK pathways can be activated under pressure load, including hypertension [11]. A wide array of studies using different experimental paradigms show pathological cardiac hypertrophy to be significantly attenuated by p38MAPK pathway inhibition [11,12,13]. p38MAPK pathway activity leads to the downstream activation of cyclic adenosine 3′,5′-monophosphate (cAMP)-response element binding protein (CREB), with CREB interacting with DNA and regulating a plethora of gene transcriptions strongly associated with LVH [14]. Brain-derived neurotrophic factor (BDNF) is also increased in LVH [15] and can contribute to LVH pathophysiology via two-way activation interactions with CREB, with increased BDNF also being induced by p38MAPK [16].

**Figure 1:**
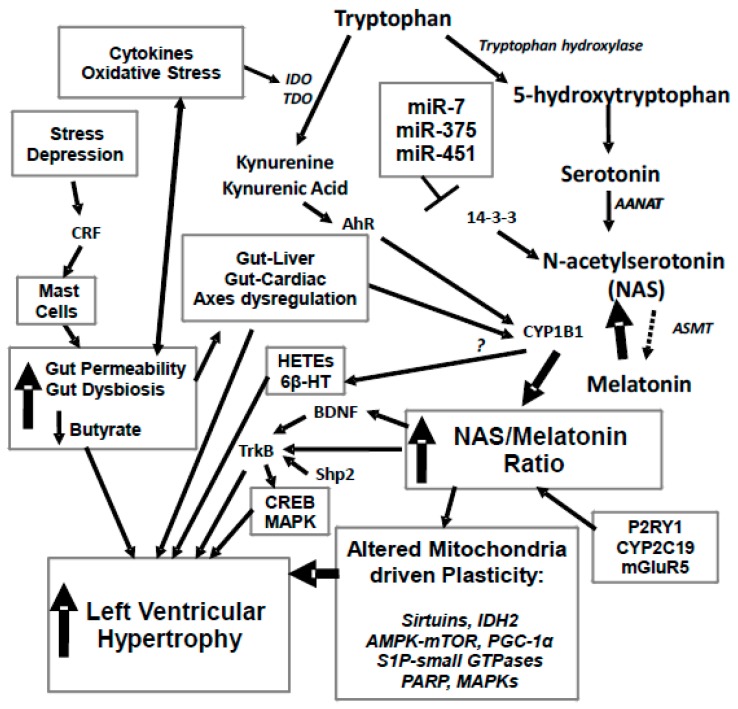
Stress and depression drive an increase in corticotropin releasing factor (CRF) that activates mucosal mast cells and leads to an increase in gut permeability, coupled to gut dysbiosis and decreased butyrate production. This drives alterations in the gut-liver and gut-cardiac axes, with a decrease in butyrate’s histone deacetylase (HDAC) inhibitory activity regulating CYP1B1. Increased gut permeability also drives an increase in oxidative stress and pro-inflammatory cytokines, which activate tryptophan 2,3-dioxygenase (TDO) and indoleamine 2,3-dioxygenase (IDO), leading to a driving of tryptophan down the kynurenine pathway, with a resultant decrease in serotonin, N-acetylserotonin (NAS) and melatonin synthesis. An increase in the NAS/melatonin ratio from CYP1B1 and probably mGluRgpI, P2Y1 receptor, CYP2C19 and O-demethylation leads to NAS activation of TrkB, as well as brain-derived neurotropic factor (BDNF) induction, in turn increasing cyclic adenosine 3′,5′-monophosphate (cAMP)-response element binding protein (CREB) and the mitogen-activated protein kinase (MAPK) pathways, which all contribute to left ventricular hypertrophy (LVH). The suppression of melatonin drives alterations in mitochondria, including via melatonin’s interactions with the sirtuins and PGC-1α, with consequences for IDH2 and AMPK-mTOR regulation of mitochondria. Alterations in the gut drive changes in the gut-liver and gut-cardiac axes that, along with kynurenine activation of the AhR raises CYP1B1 levels. CYP1B1 may also contribute to LVH via the hydroxyeicosatetraenoic acids (HETEs) and 6β-HT. A number of microRNAs known to be altered in LVH, including miR-7, miR-375 and miR-451, all act to regulate 14-3-3 and therefore the stabilization of AANAT at the start of the melatonergic pathway. Similar processes can underpin hypertension, which is the major driver of LVH. 6β-HT: 6β-hydroxytestosterone; AANAT: aralkylamine N-acetyltransferase; AhR; aryl hydrocarbon receptor; AMPK: AMP-activated protein kinase; ASMT: acetylserotonin O-methyltransferase; BDNF: brain derived neurotrophic factor; CREB: cyclic adenosine 3′,5′-monophosphate (cAMP)-response element binding protein; CRF: corticotrophin releasing factor; CYP: Cytochrome P450; E2: estrogen; GLUT1: glucose transporter 1; HDAC: histone deacetylase; HETEs: hydroxyeicosatetraenoic acids; IDO: indoleamine 2,3-dioxygenase; KAT: kynurenine aminotransferase; LVH: left ventricular hypertrophy; MAPK: mitogen activated protein kinases; mGluR: metabotropic glutamate receptor; Mito: mitochondria; NAS: N-acetylserotonin; P2Y1: purinergic receptor; PARP: poly-(ADP-ribose) polymerase; PGC-1α: peroxisome proliferator-activated receptor gamma coactivator 1-alpha; S1P: sphingosine-1-phosphate; TDO: tryptophan 2,3-dioxygenase; TrkB: tyrosine receptor kinase B. Arrows indicate activation; T-bar indicates inhibition.

A number of studies indicate a role for Src homology-2 domain-containing phosphatase2 (Shp2) in cardiac hypertrophy [17]. Shp2 is an ERK signalling mediator, with Shp2 interactions with tyrosine receptor kinase (Trk)B being necessary for BDNF to activate extracellular signal-regulated kinases (ERK) signalling [18]. It is via the ERK activation pathways and the small GTPases, RhoA and Rac1, that Shp2 acts to regulate the cellular morphology changes occurring in LVH [17]. Shp2 can then act to regulate TrkB-linked MAPK pathway activation by BDNF and other TrkB ligands, including N-acetylserotonin (NAS) [19]. NAS is the immediate precursor of melatonin in the melatonergic pathway (see Figure 1).

Overall, the MAPK pathways, including p38 MAPK and the ERKs, are strongly associated with cardiac hypertrophy. Notably, MAPK pathway activation can be driven by raised levels of CYP1B1 [20].

### 2.2. CYP1B1

Increased CYP1B1 is evident in cardiac hypertrophy, including when induced by dasatinib [21]. The increase in CYP1B1 may not necessarily be mediated via its classical induction by the AhR [21]. Rather its relevance to cardiac hypertrophy has been attributed to the mid-chain hydroxyeicosatetraenoic acids (HETEs) [20]. These authors showed that the inhibition of CYP1B1 significantly attenuates isoprenol-induced cardiac hypertrophy, with protection afforded by the modulation of superoxide anion, MAPKs, and nuclear factor-κB (NF-κB) [20], whilst the overexpression of CYP1B1 significantly induces cellular hypertrophy and mid-chain HETE metabolites. Other CYP1B1 metabolites have been shown to contribute to cardiac hypertrophy, including 6β-hydroxytestosterone [22].

Increased CYP1B1 is also relevant to the hypertensive pathoetiology of LVH, with CYP1B1 inhibition attenuating the blood pressure increase in spontaneously hypertensive rats (SHR), but not in control rodents, as well as dramatically limiting increases in vascular reactivity, cardiovascular hypertrophy, endothelial dysfunction and renal dysfunction, as well as cardiac and renal fibrosis in SHR [23]. CYP1B1 inhibition also significantly attenuates ROS production and nicotinamide adenine dinucleotide phosphate (NADPH) oxidase activity in SHR, as well as the heightened plasma levels of pro-inflammatory cytokines and catecholamines [23]. These authors also showed CYP1B1 inhibition to modulate the cardiac activity of p38 MAPK, ERK, c-Src tyrosine kinase, and Akt in SHR [23]. Such data clearly highlight a role for CYP1B1 inhibition in the management of hypertension and its associated disorders, including LVH. As such, raised CYP1B1 levels would seem relevant to both the pathoetiology and pathophysiology of LVH. CYP1B1 also ‘backward’ converts melatonin to NAS, thereby increasing TrkB activation, BDNF, CREB and the MAPKs (Figure 1). CYP1B1 levels are most commonly raised following AhR activity.

### 2.3. Aryl Hydrocarbon Receptor (AhR)

AhR activation can drive hypertrophic effects in the cardiomyocyte cell line, H9c2, concurrent to its induction of CYP1B1 in these cells [24]. Work in another cardiomyocyte cell line, AC 16 cells, shows AhR ligands to gradually raise the mRNA and protein levels of the AhR and CYP1(A1/B1), in association with attenuated mitochondrial activity, altered mitochondria membranes and heightened mitochondrial ROS levels [25]. AhR activation is associated with the development of hypertension and LVH [26]. Such data clearly shows the relevance of the AhR and CYP1B1 to the cellular and mitochondria changes occurring in LVH. The AhR induces its own repressor, the AhRR. However, not all genes activated by the AhR are inactivated by the AhRR [27], indicating that AhR activation can have longer term consequences for gene expression patterning.

It should be noted that the AhR has regulatory physiological as well as pathophysiological roles. The AhR can be activated by endogenous ligands, as well as by induced and exogenous ligands [28]. The induction of the kynurenine pathway can increase levels of kynurenine and kynurenic acid, although most work has investigated these changes in preclinical models. Both kynurenine and kynurenic acid activate the AhR and therefore CYP1B1. Heightened activation of the kynurenine pathway is evident in LVH [29]. The inclusion of the kynurenine pathways in the pathophysiology of LVH, readily allows for the inclusion of stress and affective dysregulation in the etiology of both hypertension and LVH (see Figure 1).

### 2.4. Oxidative Stress, Immune-Inflammation and Kynurenine Pathways

Heightened levels of oxidative stress and immune-inflammatory activity are evident in LVH, both systemically in clinical samples [30] and in preclinical cardiomyocytes [31], as well as in hypertension patients [32]. Consequently, the induction of oxidative stress is a preclinical model of cardiac hypertrophy [33]. Pro-inflammatory cytokines are similarly increased in hypertension [34] as well as in LVH serum and cardiomyocytes [35,36]. Low-grade inflammation, as indicated by C-reactive protein, and kynurenine pathway activation are linked to adverse cardiac remodelling [29], with raised kynurenine levels long appreciated to modulate the efficacy of antihypertensives [37]. Such systemic and local markers of oxidative stress and immune inflammatory activity are therefore likely to indicate AhR and CYP1B1 activation, as shown in preclinical models, as well as modulate the effects of medications.

The heightened levels of pro-inflammatory cytokines and oxidative stress/ROS in LVH increases the activation of indoleamine 2,3-dioxygenase (IDO) and tryptophan 2,3-dioxygenase (TDO), respectively [38]. IDO and TDO induction drives tryptophan along the kynurenine pathway and away from serotonin and melatonin synthesis [39]. Interleukin (IL)-1B, IL-6, IL-18, tumor necrosis factor (TNF)-α and interferon (IFN)-γ are increased in LVH and hypertension [40], with all these pro-inflammatory cytokines activating IDO. Likewise, oxidative stress, ROS and pro-inflammatory cytokines can induce TDO. Although IDO can regulate blood pressure [41], it has been relatively little investigated in cardiac hypertrophy. This is surprising given the increase in both IDO inducers and kynurenine pathway products in hypertension and cardiovascular disorders more widely. TDO is predominantly expressed in the liver and brain, with effects therefore more likely to be indirect [42]. This is of some importance to the role of the AhR and CYP1B1 in cardiac hypertrophy, as both kynurenine and kynurenic acid activate the AhR to increase CYP1B1.

### 2.5. Gut Dysbiosis and Gut Permeability

A plethora of recent studies have highlighted a role for alterations in the gut microbiome, in association with increased gut permeability, in driving hypertension, cardiovascular disorders, and metabolic syndrome more widely [43]. The association of psychological stress with hypertension and LVH [44] may be mediated by stress-induced corticotropin releasing factor (CRF), which acts on mucosal mast cells to increase gut permeability [45]. Gut dysbiosis and increased gut permeability are associated with the transfer of gut bacteria or tiny fragments of partially digested food over the gut barrier, leading to raised levels of circulating lipopolysaccharide (LPS), that acts on the immune system to heighten pro-inflammatory cytokines and ROS [46]. LPS activation of the toll-like receptor (TLR)4 is sufficient to drive LVH [47]. The association of chronic and repetitive stress with anxiety and depression seems at least partially mediated by gut dysbiosis and increased gut permeability [48]. Alterations in gut regulation are therefore an important aspect of how stress and affect dysregulation link to hypertension and LVH [49]. Under conditions of gut dysbiosis, there is a decrease in the microbiome-derived short-chain fatty acid, butyrate. This leads to the loss of butyrate’s protective effects on the maintenance of the gut barrier as well as on the optimization of systemic mitochondria functioning and immune dampening [46]. The latter effects are mediated by the crossing of butyrate over the gut barrier, with butyrate having optimizing effects on mitochondria regulation, at least in part via its induction of melatonin [50].

Clearly, gut dysbiosis and the gut-liver axis are important in mediating the pathophysiological processes associated with type II diabetes and the association of this condition with LVH. However, as well as dietary driven changes in the gut microbiome, the effects of psychological stress and affective dysregulation can also have their biological underpinnings in gut-mediated changes.

Overall, stress, anxiety and depression may be partly mediating their effects on hypertension and LVH, via increased gut permeability and decreased microbiome butyrate production. This increases systemic oxidative stress and pro-inflammatory cytokines that induce IDO and TDO, leading to the production of kynurenine and kynurenic acid, which activate the AhR and thereby increase CYP1B1 levels and activity that seem crucial to the pathophysiology of LVH in cardiomyocytes. The classical association of decreased serotonin in depression is strongly determined by such increases in IDO and TDO activity, including across a diverse range of medical conditions where depression is often comorbid [51], with the attenuated serotonin availability also driving down levels of melatonin and NAS. Incorporating CYP1B1 and the NAS/melatonin ratio into these gut-driven changes allows for a more plausible biological modelling of how stress and depression can link to hypertension and LVH (Figure 1).

### 2.6. Sirtuins, microRNAs, 14-3-3 and Mitochondria Functioning

CYP1B1 induction can lead to its translocation to mitochondria, where it mediates some of the toxicity associated with AhR ligands [52]. The importance of alterations in mitochondria functioning in LVH is highlighted by the data on the role of mitochondria-associated sirtuin-3 in LVH. In rodents, cardiac hypertrophy is attenuated by the inhibition of ROS and the induction of sirtuin-3 [53]. The maintenance of mitochondria sirtuin-3 attenuates both stress- and obesity-induced cardiac hypertrophy [54], highlighting the importance of sirtuin-3 and mitochondria functioning to the changes occurring in LVH. Cardiomyocyte cellular models also support a role for the loss of mitochondria sirtuin-3 in LVH [55].

Sirtuin-1 also plays a role in LVH, with sirtuin-1 alleles being risk factors for LVH in chronic kidney disease patients [56]. The efficacy of vitamin D3 against diabetes-associated cardiac hypertrophy is mediated by increased sirtuin-1, in association with attenuated DNA oxidative damage, decreased PARP1 and lower mammalian target of rapamycin (mTOR) phosphorylation [57]. As PARP1 is NAD+ dependent, it lowers the availability of the NAD+ that is necessary for sirtuin induction [6]. Protein kinase C (PKC)-ζ leads to cardiac hypertrophy via increased activity of nuclear factor-kappaB (NF-κB), ERK1/2 and ERK5, which sirtuin-1 prevents via PKC-ζ inhibition, as shown in preclinical models [58]. Sirtuin-1 is therefore a powerful regulator of many of the biological processes associated with LVH. As well as regulating mitochondria functioning via PGC-1α, which is decreased in LVH cardiomyocytes [4], sirtuin-1 can also positively regulate mitochondria via sirtuin-3. Sirtuin-1 deacetylases, and therefore positively regulates, mitochondria-associated sirtuin-3 [59]. Notably, sirtuin-3 is proposed to have co-evolved with melatonin within mitochondria, leading to their post-translational collaboration that, among other processes, acts to regulate mitochondria free radical generation and removal [60].

Sirtuin-3 also regulates mitochondria functioning and cardiac hypertrophy via the deacetylation of isocitrate dehydrogenase (IDH2). The IDH2 dimer links glucose metabolism to mitochondria oxidative phosphorylation [61]. A decrease in IDH2 activity leads to hypertrophy in cardiomyocyte cell lines in rodent LVH models [62,63], with the loss of IDH2 activity long appreciated to precede cardiac hypertrophy [64]. Sirtuin-3 is also a significant regulator of mitophagy, with dysregulated mitophagy being an important aspect of the biological underpinnings of LVH [65]. Such data highlight not only the importance of sirtuin regulation of IDH2, but also the crucial role that changes in mitochondria functioning play in the pathoetiology and pathophysiology of cardiac hypertrophy. There is a growing appreciation that mitochondria are an important hub for co-ordinating the receptor, ECM and intracellular signalling changes occurring in cells, with mitochondria driving the patterned responses and adaptations to such signalling, including in LVH [6,66].

A number of microRNAs show alterations in cardiac hypertrophy, including miR-7, miR-375 and miR-451 [67,68,69]. All three of these microRNAs can be increased in LVH and act to suppress 14-3-3 [70], with decreased 14-3-3 contributing to LVH pathophysiology [71]. It is of note that 14-3-3, miR-7, miR-375 and miR-451 can all act to regulate mitochondria functioning via alterations in the mitochondria melatonergic pathway [46]. This is mediated by 14-3-3 being necessary for the stabilization of arylalkylamine n-acetyltransferase (AANAT), which catalyses serotonin to NAS at the start of the melatonergic pathway. As such, the suppression of 14-3-3 by these miRNAs will significantly lower melatonergic pathway activity, which when coupled to increased CYP1B1 will dramatically lower melatonin availability (see Figure 1). However, it should be noted that not all studies show these three microRNAs to be increased in cardiac hypertrophy [72]. This may be a reflection on the wider dynamic and adaptive biological changes occurring in LVH, which rather than being a ‘static’ medical state may be better viewed as a series of plasticity adaptations.

### 2.7. Melatonin and LVH

A role for the melatonergic pathways in LVH is indicated by the data showing increased serotonin degradation by monoamine oxidase (MAO)A/B to be associated with LVH and wider cardiac dysregulation [73]. Serotonin is the necessary precursor of the melatonergic pathways, providing a substrate for AANAT to synthesize NAS, which is then enzymatically converted to melatonin by acetylserotonin methyltransferase (ASMT) [74]. Raised ROS and suboptimal mitochondria functioning are thought to increase MAO-A/B [75]. However, by decreasing the availability of serotonin as a substrate for the melatonergic pathways, the effects of raised MAO-A/B levels are likely to be, at least partly, mediated by the suppression of the mitochondria melatonergic pathways. Consequently, the lowered melatonin synthesis will contribute to an increase in ROS levels and suboptimal mitochondria functioning.

Given the plethora of studies showing the antioxidant, anti-inflammatory and mitochondria-optimizing effects of melatonin [50,60,66,76], a number of studies have investigated the utility of melatonin in LVH [77]. These authors showed melatonin to exert cardioprotective effects, reduce LVH remodelling and improve survival outcomes in the isoproterenol model of LVH [77]. Melatonin has clinical utility in the management of cardiac hypertrophy and affords protection in an array of different preclinical models, with effects variously attributed, including to an increase in PGC-1b [78], cyclophilin A/CD147 [79], antioxidant effects [80], retinoic acid receptor-related orphan receptor-α (RORα) [81], autophagy and AMPK [82] or a decrease in mTOR [83] and TNF-α [84]. Melatonin receptor regulating pharmaceuticals, such as ramelteon, have also shown utility in the management of LVH, suggesting a protective role for melatonin receptor activation [85].

However, it is important to note that a growing body of data shows melatonin to be produced by all mitochondria-containing cells so far investigated, usually within the mitochondria matrix [86], indicating that local melatonin synthesis and regulation may be an overlooked factor in many medical conditions [87], including glioblastoma [9], endometriosis [8] and neurodegenerative conditions [88]. The inclusion of the mitochondria melatonergic pathways in the pathophysiology of LVH allows the previously disparate bodies of data highlighted above to be integrated into a model of LVH that emphasizes the role of the mitochondria melatonergic pathways. The targeting of increased melatonin production in cardiomyocyte mitochondria may prove of far greater clinical utility than the administration of adjunctive melatonin.

## 3. Integrating LVH Pathophysiology

### 3.1. CYP1B1, AhR, Kynurenine and Melatonergic Pathway

All of the above factors can be integrated into a model of LVH that emphasizes the regulation of the mitochondria melatonergic pathways (see Figure 1). Oxidative stress and pro-inflammatory cytokines, including via gut dysbiosis and increased gut permeability, lead to IDO and TDO induction, which drives tryptophan down the kynurenine pathway and away from serotonin, NAS and melatonin synthesis. The resultant increase in kynurenine and kynurenic acid can activate the AhR, leading to the induction of CYP1B1. CYP1B1 has a number of effects that are relevant to LVH pathophyiology, including raising levels of HETEs and 6β-hydroxytestosterone. However, the strongest effects of CYP1B1 may be mediated via its ‘backward’ conversion of melatonin to NAS.

An increase in mitochondria NAS/melatonin ratio has a number of consequences for LVH. Melatonin is a powerful antioxidant and anti-inflammatory that optimizes mitochondria functioning. Melatonin can also regulate the mitochondria membrane by increasing mitochondria membrane fluidity, which is proposed to modulate the nature and interactions of the many factors that form complexes on the mitochondria membrane [6]. The AhR and CYP1(A1/B1) interact to alter mitochondria membranes [25], suggesting that the suppression of AhR effects by melatonin may include alterations in mitochondria membranes. The suppression of released mitochondria melatonin will have consequences for other cellular organelles and processes, including other mitochondria. Although NAS has some antioxidant and anti-inflammatory effects, it is also a TrkB ligand, thereby allowing NAS to mimic BDNF effects, as well as inducing an increase in BDNF mRNA and protein. Consequently, the two-way reciprocated interactions of BDNF and CREB will contribute to an increased activation of the MAPK pathways, and the trophic-type effects that this can induce. The effects of Shp2, via NAS-activated TrkB, will therefore contribute to the association of NAS with MAPK activation in LVH.

### 3.2. Sirtuins

As melatonin and sirtuin-3 seem to have long co-evolved within mitochondria, including in post-translational collaboration and mitochondria free radical regulation [60], the loss of mitochondria melatonin will have consequences for sirtuin-3 protective effects in LVH. Melatonin affords protection in many cells by increasing sirtuin 3 [89], as well as PGC-1α, which interacts with sirtuin-1 to increase sirtuin-3 and mitochondria functioning. As such, the important suppression of PGC-1α and the sirtuins in LVH may be intimately linked to the loss of mitochondria melatonin synthesis. As noted above, sirtuin-3 also regulates mitochondria functioning and LVH via its deacetylation of IDH2, and thereby impacts on the association of glucose metabolism with mitochondria oxidative phosphorylation in LVH [61,62,63]. with the loss of IDH2 activity long appreciated to precede cardiac hypertrophy [64]. Sirtuin-3, like melatonin, is an important driver of mitophagy in challenged cardiomyocytes [90], with a decrease in sirtuin-3 and melatonin contributing to the dysregulated mitophagy evident in LVH [65].

### 3.3. microRNAs

Other factors, such as the miRNAs, miR-7, miR-375 and miR-451, can act via 14-3-3 inhibition to suppress the melatonergic pathways. The inhibition of 14-3-3 by all three miRNAs will dramatically limit any melatonergic pathway activity, whilst a suppression of these miRNAs would be associated with an increased production of NAS. When coupled to an increase in CYP1B1, very little NAS would be stably converted to melatonin. As such, the alterations in these miRNAs in LVH would be likely to be associated with an increased NAS activation of TrkB and thereby increase BDNF, CREB and the MAPK pathways. The import of CYP1B1 into mitochondria may therefore be an important aspect of how mitochondria act to regulate cellular plasticity under the hypertensive conditions in LVH pathogenesis.

### 3.4. Integrating Stress and Depression

Such a model also better integrates the long-standing associations of stress, anxiety and depression with hypertension and thereby with LVH. A growing body of data shows such affective dysregulation to be mediating its effects via gut dysbiosis and increased gut permeability, leading to heightened levels of oxidative stress and immune-inflammatory activity. There is a clear link of stress/mood dysregulation to gut dysbiosis, TDO/IDO induction, raised kynurenine, AhR activation and CYP1B1 induction, which couple to a heightening of the NAS/melatonin ratio and thereby to the plethora of processes altered in LVH. Other factors that can also increase the NAS/melatonin ratio, including CYP2C19, as well as P2Y1 receptor and metabotropic glutamate receptors (mGluR) group I activation, are reviewed in [9]. CYP2C19, mGluR5 and P2Y1 receptors are also associated with stress and depression [91,92,93], as well as with hypertension [94,95,96]. Such data would suggest that alterations in the regulation of the melatonergic pathways may also be relevant to stress/depression and hypertension, and therefore in the pathoetiology of LVH. Clearly, alteration in the mitochondria melatonergic pathway is an important aspect of plasticity and adaptive responses in all cells and cellular interactions. The high density of mitochondria within cardiomyocytes would make this particularly relevant in these cells.

### 3.5. Gut Microbiome, Butyrate and Histone Deacetylation

As indicated above, there is a growing realization as to the importance of the gut in a plethora of medical conditions, especially in regard to metabolic syndrome and obesity, where roles for the gut-liver and gut-cardiac axes are particularly important. Lower levels of the microbiome-derived short-chain fatty acid, butyrate, can lead to significant changes in these axes. Butyrate seals the gut barrier, thereby preventing the leakage of LPS and food fragments that drive the heightened oxidative stress and inflammatory activity associated with increased gut permeability and with the plethora of LVH-associated changes that follow, as indicated above. Clearly this is important to the functioning of the gut-liver and gut-cardiac axes. Butyrate can also cross intestinal epithelial cells into the general circulation, where it acts to dampen immune responsivity and better optimize mitochondria functioning [46]. Some of the effects of butyrate may be mediated by its induction of melatonin [50].

However, most of the effects of butyrate are mediated via histone deacetylase (HDAC) inhibition, with consequences for AhR-induced CYP1, as shown in intestinal epithelial cells [97]. The relevance of butyrate’s HDAC inhibitory activity regarding AhR-induced CYP1 in cardiomyocytes requires investigation. The commercially available salt version of butyrate, sodium butyrate, negatively regulates cardiac hypertrophy in rodents via HDAC inhibition [98]. Activating transcription factor 3 (ATF3) in cardiac fibroblasts, via HDAC inhibition, suppresses the p38MAPK pathway, thereby limiting cardiac hypertrophy [99]. Sodium valproate, another HDAC inhibitor, can also suppress LVH in preclinical models [100]. Interestingly, sodium valproate also increases melatonin MT1 receptors and possibly melatonin synthesis, as shown in astrocytes [101]. As such, HDAC inhibition by sodium valproate or butyrate can be associated with melatonin synthesis and receptor upregulation, which requires investigation in cardiomyocytes. Overall, the gut is an important hub for co-ordinating the changes occurring in the etiology and pathophysiology of LVH, with butyrate likely to have impacts on LVH via HDAC inhibition, the melatonergic pathways and mitochondria functioning.

### 3.6. Exosomes

Recent studies have highlighted a role for exosomes in the pathophysiology and possible treatment of LVH [102,103]. Although many factors can be carried by exosomes, miRNAs are thought to be the most important to changing the patterned gene expressions relevant to LVH. Such exosome cargoes can be local, as with cardiac fibroblast exosomal miRNAs being incorporated into cardiomyocytes [104], or more distant, as with intestinal epithelial cell exosomes targeting the immune system [105] or brain neuronal functioning [106]. As both butyrate and melatonin can modulate the content of exosomes [107,108], it is clear that the butyrate effects on intestinal epithelial cell exosomes and the impact of these exosomes, directly or indirectly, on distant target sites such as cardiomyocytes will be important to clarify, including as to their impact on the regulation of the melatonergic pathways and mitochondria functioning.

### 3.7. Epigallocatechin-3-Gallate

Such a model also provides a conceptual framework for reattributing the biological underpinnings of an array of factors that are known to modulate LVH. For example, green tea’s epigallocatechin-3-gallate (EGCG) has been widely reported to decrease LVH [109]. The effects of EGCG have been variously attributed to the regulation of MAPKs, AMPK, antioxidants, telomere maintenance, NF-κB inhibition, decreased glucose in diabetes and Hippo signalling, reviewed in [110]. However, EGCG also modulates the gut microbiome, gut permeability and butyrate levels [111] as well as decreasing MAO-B [112], suggesting that it would have an impact on the levels of serotonin available as a substrate for the melatonergic pathway. EGCG also inhibits the AhR [113] and IDO as well as having antioxidant and anti-inflammatory effects that would all contribute to suppressing the driving of tryptophan down the kynurenine pathway [114]. Although clearly requiring direct investigation, such data would suggest that EGCG would have impacts in the gut and the various gut axes, as well as on the regulation of the melatonergic pathways, including possibly in cardiomyocytes.

## 4. Research and Treatment Implications

Given the associations of stress, depression, obesity and hypertension in the pathoetiology and pathophysiology of LVH, it is clear that an array of biological systems interacting over time add a considerable complexity to an understanding of its biological underpinnings. The above would suggest that an array of previously disparate bodies of data on LVH may be linked to the regulation of the melatonergic pathways within mitochondria. Although melatonin production has been shown in an array of different cells, including epithelial cells, glia, neurons, immune cells and every other mitochondria-containing cell so far investigated, the melatonergic pathways have not been investigated in cardiomyocytes. This will be important to rectify, given the considerably higher levels of mitochondria in these cells.

Many of the factors that are proposed to modulate the melatonergic pathway in mitochondria, including CYP1B1, the AhR, 14-3-3, melatonin, miR-7, miR-375, miR-451, BDNF/TrkB, CYP2C19, mGluRgpI, P2Y1 receptors and O-demethylation [9,70] are all involved in the regulation of cardiac hypertrophy, as indicated above. This could suggest a role for the regulation of the mitochondria melatonergic pathway in LVH. The impact of these factors on the NAS/melatonin ratio and the relevance of this ratio in modulating mitochondria-driven cell processes, including via miRNA and gene patterning, will be important to determine. This is not unlikely to provide novel treatment targets.

The relevance of gut dysbiosis to the pathoetiology of LVH requires further investigation, including as to the roles of increased gut permeability and intestinal epithelial cell exosomes, which may be co-ordinated with decreases in microbiome-derived butyrate. Given that the gut-cardiac and gut-liver axes are etiologically important to obesity, type II diabetes and hypertension, it would seem clear that the gut may be intimately linked to the pathoetiology of LVH. Targeting gut dysbiosis, gut permeability and increased microbiome-derived butyrate is likely to have clinical utility at all stages of LVH pathoetiology and pathophysiology.

Sodium butyrate, via HDAC inhibition, can prevent LVH in preclinical models. The use of sodium butyrate also increases the presence of butyrate-producing gut bacteria, further strengthening the case for its use in the treatment of LVH and its etiological risk factors. The relevance of dietary sodium butyrate to direct and/or indirect effects on cardiomyocytes will be important to determine, including as to its impact on intestinal epithelial cell exosomal content. As sodium butyrate increases melatonin synthesis, as shown in intestinal epithelial cells [50], it will be important to clarify as to whether melatonin is mediating some of the effects attributed to butyrate.

As noted, sirtuin-3 and melatonin are significant regulators of mitophagy in challenged cardiomyocytes [90], with mitophagy dysregulated in LVH [65]. Recent work indicates that pineal melatonin can increase the levels and activity of the mitochondria melatonergic pathway, allowing variations in pineal gland-derived, night-time melatonin to regulate NAS and melatonin production in mitochondria [115]. The uptake of exogenous or pineal gland-derived melatonin into mitochondria, as well as the melatonin produced within mitochondria, may contribute to mitophagy in cardiomyocytes, including via melatonin-induced sirtuin-3. This will be important to determine in cardiomyocytes as this could indicate a role for alterations in pineal circadian melatonin in the regulation of mitophagy in cardiomyocytes, at least in part via the regulation of the mitochondria melatonergic pathway. Clearly, increased AhR activation and CYP1B1 induction in mitochondria would act to inhibit such melatonin effects on mitophagy [116]. Some of the effects of melatonin in mitochondria are mediated via its regulation of pyruvate dehydrogenase kinase and therefore the pyruvate dehydrogenase complex [115]. It is of note that pyruvate dehydrogenase kinase is a significant modulator of mitophagy [117]. Overall, the role of melatonin in mitochondria requires investigation in cardiomyocytes, especially as to how melatonin interacts with the array of changes occurring in cardiac hypertrophy, including in the regulation of mitophagy.

A number of immediately applicable clinical implications arise from the above integration. As indicated, the utilization of sodium butyrate may provide a more rapid change in the availability of gut butyrate, compared to long-term probiotic use. However, probiotics and changes in diet to encourage the growth of butyrate-producing gut bacteria are also likely to have utility in the management of LVH, as well as having wider health benefits. Although some of the effects of butyrate can be mediated via an increase in the activation of the melatonergic pathways, it is likely that the addition of melatonin would afford further benefit. As indicated above [115,116], the loss of pineal gland-derived circadian melatonin in metabolic disorders, and ageing more generally, will contribute to alterations in the mitochondria of cardiomyocytes. The use of melatonin shortly before bed should compensate such pineal gland melatonin loss. A number of other potential clinical targets, such as inhibiting mitochondria CYP1B1, require further investigation.

## 5. Conclusions

Incorporating the mitochondria melatonergic pathway in LVH better integrates a wide array of previously disparate data, including as to how CYP1B1 and the sirtuins are so strongly associated with LVH pathophysiology. Given the high levels of mitochondria in cardiomyocytes, it is urgently requiring investigation as to the presence, and relevance, of the melatonergic pathway in cardiomyocyte mitochondria. As indicated above, many of the factors associated with LVH pathophysiology may be linked via their regulation of the mitochondria melatonergic pathway. An increase in the NAS/melatonin ratio allows NAS to activate TrkB and the CREB and MAPK pathways. Gut dysbiosis is likely to be an intimate aspect of both the pathoetiology and pathophysiology of LVH, with sodium butyrate, via HDAC inhibition, being an immediately applicable treatment for all stages of LVH development.

## Abbreviations

6β-HT	6β-hydroxytestosterone
AANAT	aralkylamine N-acetyltransferase
AhR	aryl hydrocarbon receptor
AMPK	AMP-activated protein kinase
ASMT	acetylserotonin O-methyltransferase
BDNF	brain derived neurotrophic factor
CREB	cyclic adenosine 3′,5′-monophosphate (cAMP)-response element binding protein
CRF	corticotrophin releasing factor
CYP	Cytochrome P450
E2	estrogen
EGCG	epigallocatechin gallate
GLUT1	glucose transporter 1
HDAC	histone deacetylase
HETEs	hydroxyeicosatetraenoic acids
IDH	isocitrate dehydrogenase
IDO	indoleamine 2,3-dioxygenase
IL	interleukin
KAT	kynurenine aminotransferase
LVH	left ventricular hypertrophy
MAO	monoamine oxidase
MAPK	mitogen activated protein kinases
mGluR	metabotropic glutamate receptor
miR	microRNA
Mito	mitochondria
mTOR	mammalian target of rapamycin
NAD	nicotinamide adenine dinucleotide
NAS	N-acetylserotonin
P2Y1	purinergic receptor
PARP	poly-(ADP-ribose) polymerase
PGC-1α	peroxisome proliferator-activated receptor gamma coactivator 1-alpha
ROS	reactive oxygen species
S1P	sphingosine-1-phosphate
TDO	tryptophan 2,3-dioxygenase
TLR	toll-like receptor
TNF	tumor necrosis factor
TrkB	tyrosine receptor kinase B
